# Influence of Bi_2_O_3_ on Mechanical Properties and Radiation-Shielding Performance of Lithium Zinc Bismuth Silicate Glass System Using Phys-X Software

**DOI:** 10.3390/ma15041327

**Published:** 2022-02-11

**Authors:** Aljawhara H. Almuqrin, M. I. Sayyed, Nimitha S. Prabhu, Sudha D. Kamath

**Affiliations:** 1 Department of Physics, College of Science, Princess Nourah bint Abdulrahman University, P.O. Box 84428, Riyadh 11671, Saudi Arabia; ahalmoqren@pnu.edu.sa; 2 Department of Physics, Faculty of Science, Isra University, Amman 11622, Jordan; 3 Department of Nuclear Medicine Research, Institute for Research and Medical Consultations (IRMC), Imam Abdulrahman bin Faisal University (IAU), Dammam 31441, Saudi Arabia; 4 Department of Physics, Manipal Institute of Technology, Manipal Academy of Higher Education, Manipal 576104, Karnataka, India; nimprabhu14@gmail.com (N.S.P.); sudhakamath6@gmail.com (S.D.K.)

**Keywords:** bismuth silicate glasses, Makishima–Mackenzie model, gamma radiation, radiation protection, attenuation factors

## Abstract

We analyzed the mechanical properties and radiation-shielding performance of a lithium zinc bismuth silicate glass system. The composition of these glasses is 20ZnO-30Li_2_O-(50-x)SiO_2_-xBi_2_O_3_ (x varies between 10 and 40 mol%). The mechanical properties of the investigated glass system, such as Young’s modulus (*E*), bulk modulus (*K*), shear modulus (*S*), and longitudinal modulus (*L*), were determined using the Makishima–Mackenzie model. The elastic moduli gradually decreased with the addition of Bi_2_O_3_. *E* decreased from 46 to 31 GPa, *K* decreased from 27 to 14 GPa, *S* decreased from 19 to 14 GPa, and *L* decreased from 52 to 32 GPa as Bi_2_O_3_ was substituted for SiO_2_. The mass attenuation coefficient (MAC) was investigated at energies between 0.284 and 1.33 MeV to understand the radiation-shielding performance of the glasses. The MAC value increased when SiO_2_ was replaced by Bi_2_O_3_. We found that the effect of Bi_2_O_3_ on MAC values was noticeably stronger at energies of 0.284 and 0.347 MeV, while the effect of Bi_2_O_3_ on MAC values became weaker as energy increased. The linear attenuation coefficient (LAC) results demonstrated that if the samples were exposed to low-energy photons, the glass could prevent the penetration of photons, and thus, the glass samples were effective in radiation protection. The LAC values for the lowest- and highest-density samples changed from 0.998 to 1.976 cm^−1^ (at 0.284 MeV) and from 0.286 to 0.424 cm^−1^ (at 0.826 MeV). According to the radiation-shielding results, the thick, high-density glass sample has special and distinctive shielding properties.

## 1. Introduction

Recently, the use of ionizing radiation has increased in daily activities and has been introduced to many applications, including medical, agricultural, engineering, industrial, and many other fields [1,2,3,4,5]. However, it is known that excessive exposure to this radiation results in many negative effects on humans, including various diseases, cancers, and blood cell damage. Sometimes, long-term exposure to certain types of radiation may cause death. Consequently, it has become necessary to protect humans and their environments from the negative effects of radiation by designing certain types of materials that have the ability to absorb radiation and thus to reduce its impact on humans [6,7,8,9]. Many researchers have studied the radiative attenuation properties of different materials, and based on the obtained results, they have concluded that lead and concrete are among the most effective materials in this field. This is due to the high densities of lead and concrete and, thus, their high ability to attenuate photons and reduce their intensity. Despite their strong ability to attenuate photons, these materials have some disadvantages that limit their use in some practical applications. These disadvantages are due to the heavy weight of lead, which renders it difficult to use as an apron, as it will strain the spine; in addition, lead and concrete are opaque (nontransparent) materials, rendering it impossible to use these materials in the development of face shields or eye protection. Most importantly, the toxicity of lead has led to its classification as a dangerous material with negative environmental effects [10,11]. However, it is worth mentioning that glass is among the most promising materials that have proven effective in radiation shielding [12,13,14]. This is due to its important physical properties, such as high transparency, ease of preparation, low manufacturing cost, high density, and the ability to control its density and effective atomic number by incorporating certain heavy elements [15,16,17]. One of the most famous types of glass that is widely used in multiple applications is glass containing a high proportion of $B_{2}O_{3}$, called borate glass. This type of glass has good thermal stability, low viscosity, good luminous properties, and high transparency [18,19]. It is known that the density of $B_{2}O_{3}$ is low (less than 3 g/${\mathrm{cm}}^{3}$), and thus, borate glasses have a lower density compared with lead oxide glasses. Certain heavy metal oxides (HMOs), such as ${\mathrm{Bi}}_{2}O_{3}$, PbO, and ${\mathrm{TeO}}_{2}$, among others, should be used to increase the density of borate glasses [19,20]. The use of these oxides leads to an increase in the effective atomic number of the resulting glass, and thus, this glass can be used in nuclear radiation protection applications. In previous years, a group of researchers developed different types of borate glasses with HMOs and evaluated the radiation-shielding properties of the resulting glass systems [21,22,23]. There are some physical quantities that must be determined when studying the radiation protection properties of any material, which is undertaken in several ways, the most important of which is simulation. Simulation is a method that saves researchers much time and effort by using a computer to form the required observations without having to obtain them experimentally and without biasing the obtained results or their accuracy. One of the most important and most common simulation methods in radio physics is the Monte Carlo method [24,25]. For example, Al-Hadeethi et al. [26] used Geant4 to study the radiative attenuation factors of borate glasses containing ${\mathrm{WO}}_{3}–$ZnO–PbO. According to the results obtained, increasing the proportion of ${\mathrm{WO}}_{3}$ and decreasing the proportion of ZnO led to an increase in radiation protection efficiency (RPE) values, which means an improvement in the radiation protection properties. Al-Buriahi and Y.S. Rammah [27] were able to calculate the radiative attenuation factors for ZnO$–{\mathrm{TeO}}_{2}–$PbO glass using Geant4. The researchers validated the simulation results by comparing them with the results obtained using WinXCom and found an acceptable agreement between the simulation results and the theoretical results. Cheewasukhanont et al. [28] empirically measured the radiative attenuation factors of ${\mathrm{WO}}_{3}–{\mathrm{Gd}}_{2}O_{3}–B_{2}O_{3}$ glass and compared the empirical results with the results obtained using Geant4. The researchers found that ${\mathrm{WO}}_{3}–{\mathrm{Gd}}_{2}O_{3}–O_{3}$ glass had a lower half-value layer (HVL) than some types of conventional glass as well as some types of concrete. Boukhris et al. [29] calculated the radiative attenuation factors for tellurite–lead–tungsten glasses using Geant4 and found a direct relationship between the quantity of ${\mathrm{TeO}}_{2}$ and ${\mathrm{WO}}_{3}$ in the glass and the material’s ability to attenuate photons. To complement previous research in this field, we studied the radiative attenuation factors of a bismuth-containing lithium zinc silicate glass system, which was previously investigated by S. Rani et al. [30] to explore the influence of Bi_2_O_3_ on its thermal, physical, and dielectric properties. Herein, we continue the study of the influence of Bi_2_O_3_ on the mechanical and radiation-shielding properties of this glass system.

## 2. Methodology

The composition of the glass system was investigated by S. Rani et al. [30] to explore its thermal, physical, and dielectric properties, and the associated values are given in Table 1. The glass system was chosen here because it contains the heavy metal oxide Bi_2_O_3_. Moreover, the authors had already varied the content of Bi_2_O_3_ from 10 to 40 mol%, and because of this, we were interested in investigating the role of Bi_2_O_3_ in the alteration of radiation attenuation features.

The investigated glasses were previously prepared using the melt-quenching technique, as discussed in [30]. The authors used the following chemicals in the preparation of the samples: SiO_2_, Bi_2_O_3_, ZnO, and Li_2_CO_3_. They mixed these chemicals in an alumina crucible and melted them in air using an electric muffle furnace for 1 h. The melting temperature varied between 1100 and 1200 °C. The glass samples were then annealed for about 24 h at 300 °C. Moreover, these glasses exhibited a wide transmission in the visible and near-infrared regions and high thermal stability [30]. Such features would be beneficial for transparent radiation-shielding applications. However, as the radiation-shielding application requires not only the knowledge of some radiation attenuation parameters but also the rigidity and strength of the glasses, the mechanical properties were investigated in this study. Using the Makishima–Mackenzie model [31], the packing density and the mechanical properties were determined. For radiation-shielding properties, we used Phys-X software to determine the attenuation factors [5].

## 3. Results and Discussion

### 3.1. Mechanical Properties

**Figure 1:**
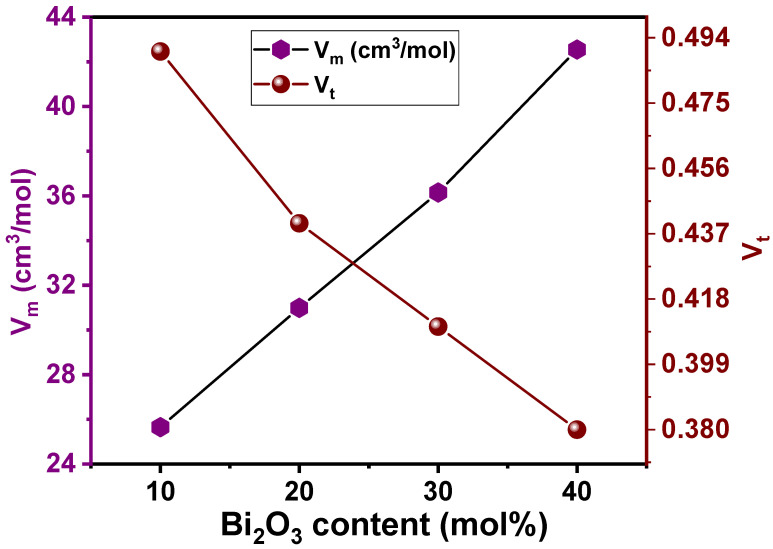
Trends in V_m_ and V_t_ with addition of Bi_2_O_3_ in the LZBS glass system.

S. Rani et al. [30] observed that the density (ρ in Table 1) increased with Bi_2_O_3_ content. A continuous rise in ρ values from 3.74 to 5.12 g/cm^3^, as seen in Table 1, was noticed when Bi_2_O_3_ replaced SiO_2_. The authors [30] associated the increasing density values with the replacement of lighter SiO_2_ with heavier Bi_2_O_3_. The trend in molar volume (V_m_) of the glass system with Bi_2_O_3_ content is presented with the values in Table 1 and plotted in Figure 1. Values for V_m_ continuously increased from 25.65 to 42.55 cm^3^/mol when Bi_2_O_3_ was substituted for SiO_2_. This indicates the loosening of the structure, which was attributed to the inability of the voids of the silicate network to accommodate Bi^3+^ ion modifiers without any expansion of the glass matrix [30]. Considering the V_m_ values, the packing density (V_t_) of the LZBS glass system was determined using the Makishima–Mackenzie model [31]. S. Rani et al. [30] reasoned that the larger ionic radius and bond length in Bi_2_O_3_ compared to those in SiO_2_ could have caused the formation of excess free volume, leading to the increase in the overall volume of the glass system. The same interpretation can be applied to decreasing V_t_ values (from 0.49 to 0.38), as seen in Figure 1. Figure 1 also demonstrates that the trends in the variation of V_m_ and V_t_ were opposite.

**Figure 2:**
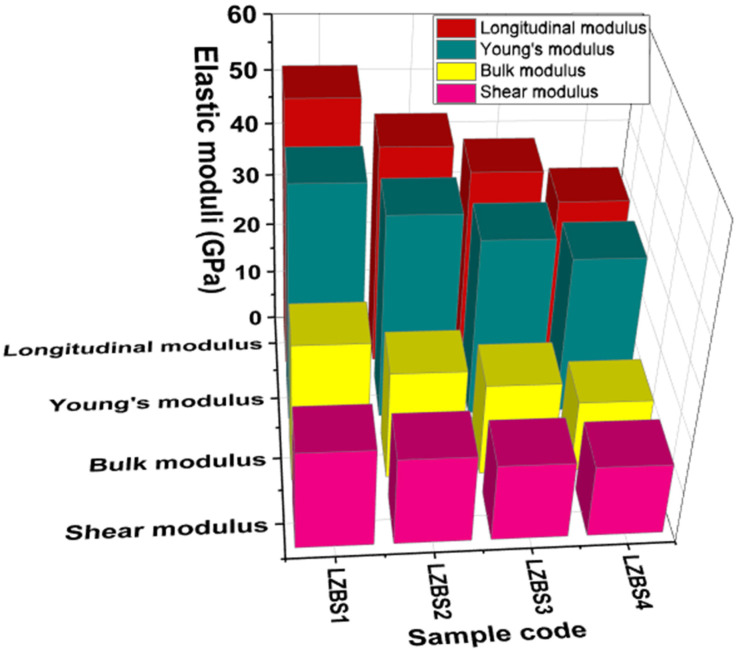
Trends in the longitudinal, Young’s, bulk, and shear moduli of the LZBS glass system.

The mechanical properties of the LZBS glass system were obtained using the Makishima–Mackenzie model [31]. This model is based on the strength of the chemical bonds or the dissociation energy per unit volume (G_t_) of the chemical compounds and V_t_. The G_t_ values were acquired from the dissociation energy per unit volume of the ith chemical component through its density, molecular weight, and molar heat of formation by the relation in [31] and data in [32]. With the necessary relations in [31], the elastic moduli were computed. The values are presented in Table 1, and the trends in their variation for each glass are depicted in Figure 2. The elastic moduli gradually decreased with the addition of Bi_2_O_3_. *E* decreased from 46 to 31 GPa, *K* decreased from 27 to 14 GPa, *S* decreased from 19 to 14 GPa, and *L* decreased from 52 to 32 GPa as Bi_2_O_3_ was substituted for SiO_2_. The hardness parameter [33] also decreased from 3.55 to 3.32 GPa with the successive addition of Bi_2_O_3_. The elastic modulus trend reflects a decline in the network cross-linking or connectivity, which is in line with the trends in V_m_ and V_t_.

### 3.2. Radiation-Shielding Properties

**Figure 3:**
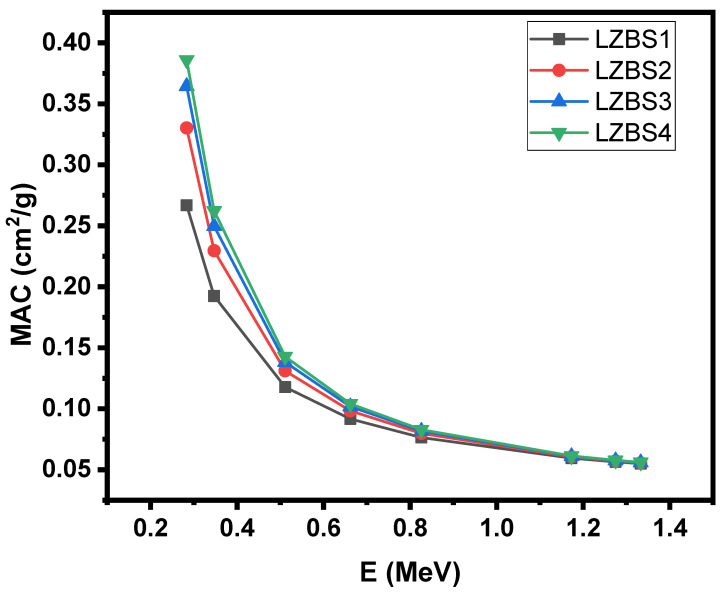
Trends in the mass attenuation coefficient of the LZBS glass system.

Figure 3 presents the graphical relationship between each mass attenuation coefficient (MAC) for the selected glasses and the energy. The energy was chosen within the range from 0.284 to 1.33 MeV, and theoretical calculations were performed using PSD software. Figure 3 shows that the MAC values depend heavily on the concentration of Bi_2_O_3_ as well as on the photon energy, as the MAC values for all glass samples decreased as the photon energy increased from 0.284 to 1.33 MeV, while the MAC value increased when SiO_2_ was replaced by Bi_2_O_3_. The LZBS1 sample contained the least amount of Bi_2_O_3_ (the highest concentration of SiO_2_) and, thus, had the lowest MAC, whereas the concentration of Bi_2_O_3_ increased from the LZBS1 sample to the LZBS4 sample, causing an increase in MAC values, with the highest value determined for the glass containing the highest percentage of Bi_2_O_3_. The reason for this increase is that the atomic number of Bi is higher than that of Si, so increasing the concentration of Bi_2_O_3_ improves the MAC value of the samples under study. Figure 3 also shows that the effect of Bi_2_O_3_ on MAC values was noticeably stronger at energies of 0.284 and 0.347 MeV and that the effect of Bi_2_O_3_ on MAC values became weaker as the energy increased. At 0.284 MeV, the MAC values of the LZBS1 and LZBS4 samples changed from 0.267 to 0.386 cm^2^/g (the difference was 0.119), whereas the difference in MAC values for the same two samples but at an energy level of 0.826 MeV was only 0.006. The reason for this is due to the photoelectric effect, which is very important at low energies, and it is well known that this effect depends inversely on energy and heavily depends on the atomic number [34]. As shown in Figure 3, the LZBS4 sample had the highest MAC.

**Figure 4:**
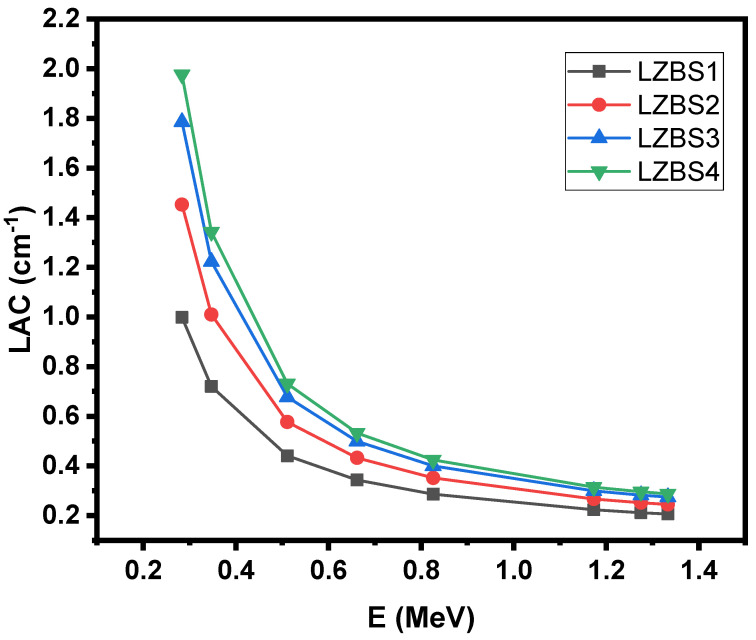
Trends in the linear attenuation coefficient of the LZBS glass system.

We also show the relationship between the linear attenuation coefficient (LAC) of the samples and energy (see Figure 4) to understand the effect of sample density on the efficiency of the samples in radiation shielding. We noticed an inverse relationship between LAC and energy, and this result is the same as in the previous figure, indicating that the radiation-shielding efficiency of glass samples is clearly dependent on the radiation energy. Therefore, if the samples are exposed to low-energy photons, the glass can prevent photons from penetrating it, and thus, the glass samples are effective in radiation protection; however, as the energy of the photons increases, the ability of the samples to attenuate the photons decreases, and their efficiency becomes low. In other words, glass is extremely effective at attenuating low-energy rays. Figure 4 shows that energy is not the only factor that influences the efficiency of glass in attenuating the photons; there is also the density of the glass to consider. As we mentioned when discussing the mechanical properties of these samples, changing the glass composition (by changing the concentrations of SiO_2_ and Bi_2_O_3_) causes a clear change in the density, which results in a noticeable change in the LAC values. For example, if we compare the LAC values for the lowest-density sample (LZBS1) and the highest-density sample (LZBS4), we notice that the value changes from 0.998 to 1.976 cm^−1^ (at an energy level of 0.284 MeV), from 0.720 to 1.343 cm^−1^ (at 0.347 MeV), and from 0.286 to 0.424 cm^−1^ (at 0.826 MeV). Thus, high-density samples have a positive effect on the shielding of materials, and the higher the density of the glass, the better the radiation-shielding effect. This figure emphasizes the significance of manufacturing high-density glass to obtain a material capable of effective radiation protection.

**Figure 5:**
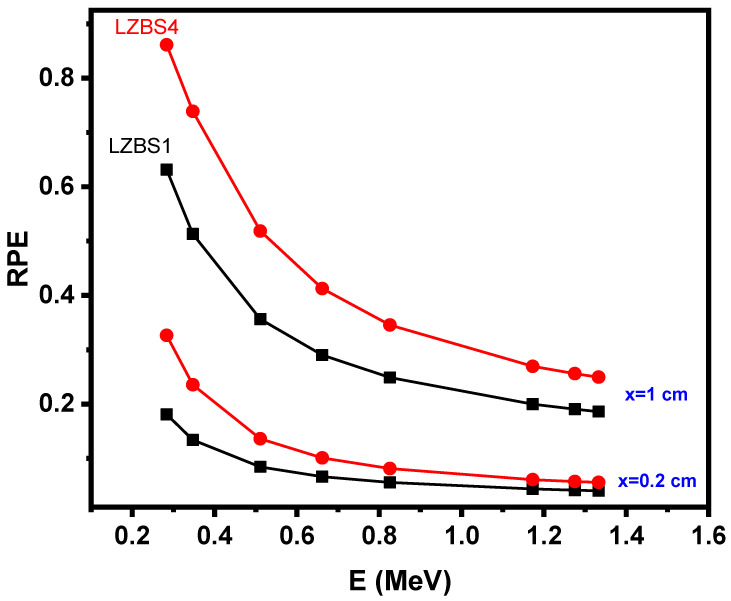
Trends in the radiation protection efficiency (RPE) of LZBS1 and LZBS4 glasses at 0.2 and 1 cm.

From the abovementioned figures (i.e., Figure 3 and Figure 4), we were able to determine the effects of energy, density, and concentrations of SiO_2_ and Bi_2_O_3_ on the radiation-shielding properties of the glass system under study. We calculated the radiation protection efficiency (RPE) of the LZBS1 and LZBS4 samples at two thicknesses (i.e., 0.2 and 1 cm) to understand the effect of the thicknesses of the samples on improving radiation-shielding properties, and the results are presented in Figure 5. From the figure, we notice that the greater the increase in the thickness of the sample, the greater the increase in the RPE value, which gives a clear indication that the shielding properties were improved by increasing the thickness of the glass sample. When we applied the rule to the LZBS4 sample, we noticed that the RPE value changed from 32 to 86% when changing the sample’s thickness from 0.2 to 1 cm (at the first energy level). When we changed the energy level of the same sample to 0.347 MeV, we noticed that the value of RPE improved from 23 to 73% when changing the sample’s thickness from 0.2 to 1 cm, which indicates a need to use a thick layer of glass to achieve photon attenuation. When considering the value of RPE at a certain thickness, we noticed that the RPE value of the LZBS4 sample was higher than that of the LZBS1 sample, which suggests that the sample that contains a large amount of Bi_2_O_3_ is more efficient and effective in radiation protection than that which contains 10 mol% of Bi_2_O_3_ (LZBS1). This result confirms the importance of using Bi_2_O_3_ to improve the radiation-shielding competence of the glass. According to the results shown in the abovementioned three figures, it can be argued that the thick, high-density glass sample has special and distinctive shielding properties, as it can be applied in practical applications specialized to protect humans from ionizing radiation.

**Figure 6:**
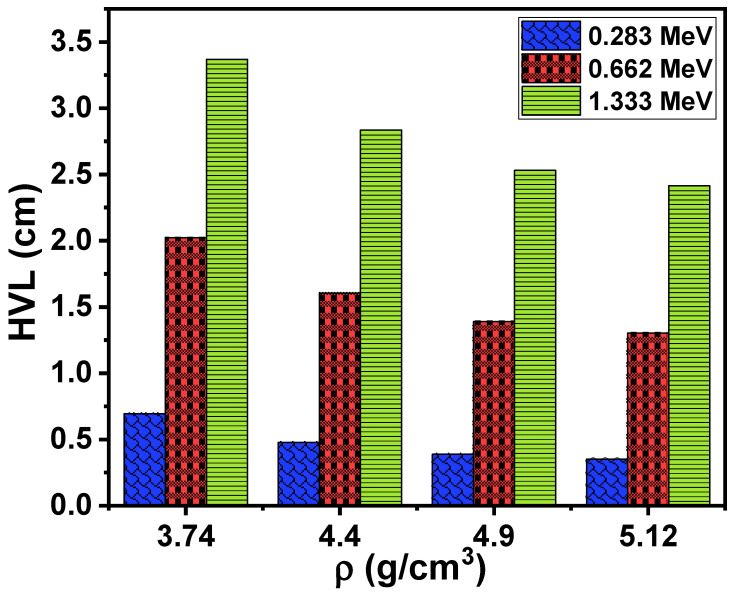
Trends in the half-value layer for the LZBS glass system.

We calculated the half-value layer (HVL) to determine the effect of the composition of the samples as well as the density of the samples’ thicknesses required to attenuate 50% of the intensity of the original photons. The relationship between HVL and the density of the samples is displayed in Figure 6. We notice from the figure that the greater the increase in the energy of the photons, the greater the increase in the HVL. Accordingly, energy is considered the first factor that affects the thickness of the samples required to absorb 50% of the photons emitted by the radioactive source. Photons with low energy will require a thin sample of glass to become attenuated. For example, the HVL values in this study ranged between 0.351 and 0.694 cm at the lowest energy, and when the energy was increased to 0.662 MeV, the HVL values increased to a range between 1.302 and 2.022 cm. Energy is not the only factor that affects the HVL. In reviewing the HVL values of the glass samples at a certain energy, we notice that these values changed when the chemical composition of the samples changed (it changed with the change in density). We also notice that the LZBS1 sample had the highest HVL value and decreased with the addition of Bi_2_O_3_, reaching the lowest value for the sample that contained the highest concentration of Bi_2_O_3_, which means that adding Bi_2_O_3_ reduces the sample dimensions needed to attenuate the photons. Therefore, the use of glass samples containing a high concentration of Bi_2_O_3_ will be appropriate, as the dimensions of the samples are small in this case. The HVL ranged between 0.351 and 2.344 cm for the highest-density sample (LZBS4).

**Figure 7:**
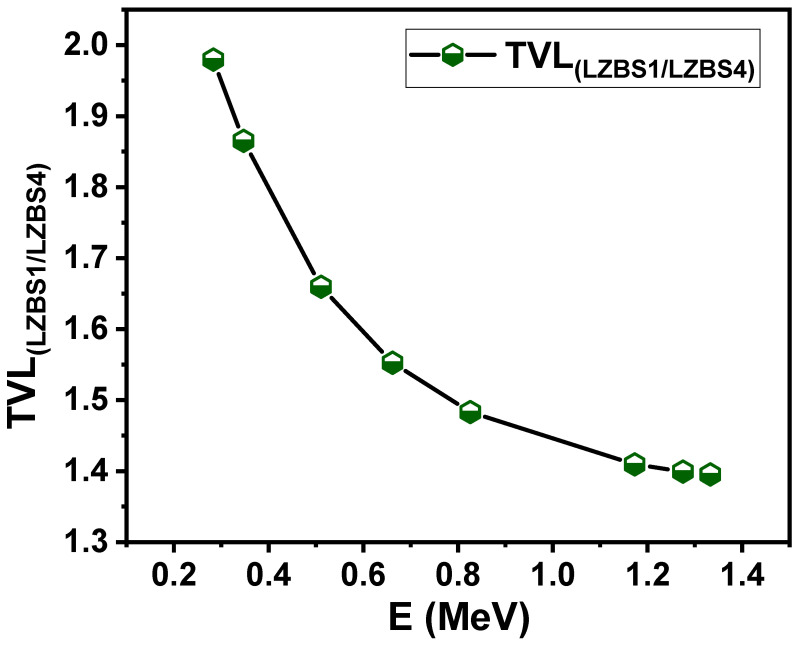
Ratios between the tenth-value layer for LZBS1 and LZBS4 samples.

The tenth-value layer (TVL) value and the ratio between TVL for the LZBS1 and LZBS4 samples were calculated, and the results are presented in Figure 7. The aforementioned samples were chosen because they contained the lowest and the highest concentrations of Bi_2_O_3_. From Figure 7, we notice that the ratio is higher than 1, which is true at all energies, which means that the TVL value of the LZBS1 sample is higher than that of the LZBS4 sample. This result agrees with the previous figure and emphasizes the importance of using Bi_2_O_3_ to improve the radiation-shielding properties of the prepared glass. We also note that the ratio was highest at the first energy (1.97), and it began to decrease as the energy increased; then, it decreased to 1.86 at an energy of 0.347 MeV, decreased again to 1.66 at an energy of 0.511 MeV, and continued to decrease to 1.55 at an energy of 0.662 MeV until it reached its lowest value of 1.39 at an energy of 1.33 MeV.

**Figure 8:**
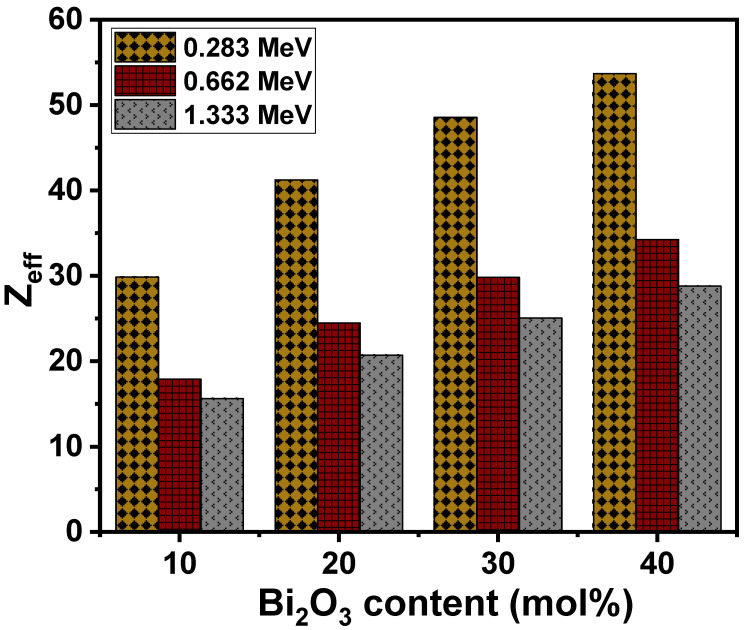
Trends in the effective atomic number of the LZBS glass system.

In Figure 8, the effective atomic number (Z_eff_) of the glasses is shown for the lowest and highest energies of 0.283 and 1.333 MeV used in this study. It is observed that Z_eff_ declined as the energy increased. The value of Z_eff_ was between 29.86 and 53.69 for the LZBS1 and LZBS4 samples at 0.283 MeV, and at 1.333 MeV, the values ranged from 15.62 to 28.82. This is due to the interplay of the photoelectric effect and Compton scattering. Moreover, Z_eff_ gradually increased as Bi_2_O_3_ content increased from 10 to 40 mol% for a given energy. This indicates a higher photon-interaction probability at higher concentrations of Bi_2_O_3_. The observation that the LZBS4 glass had the highest Z_eff_ values at a given energy implies that it is more able to block the incident radiation when compared with glasses with lower contents of Bi_2_O_3_.

**Figure 9:**
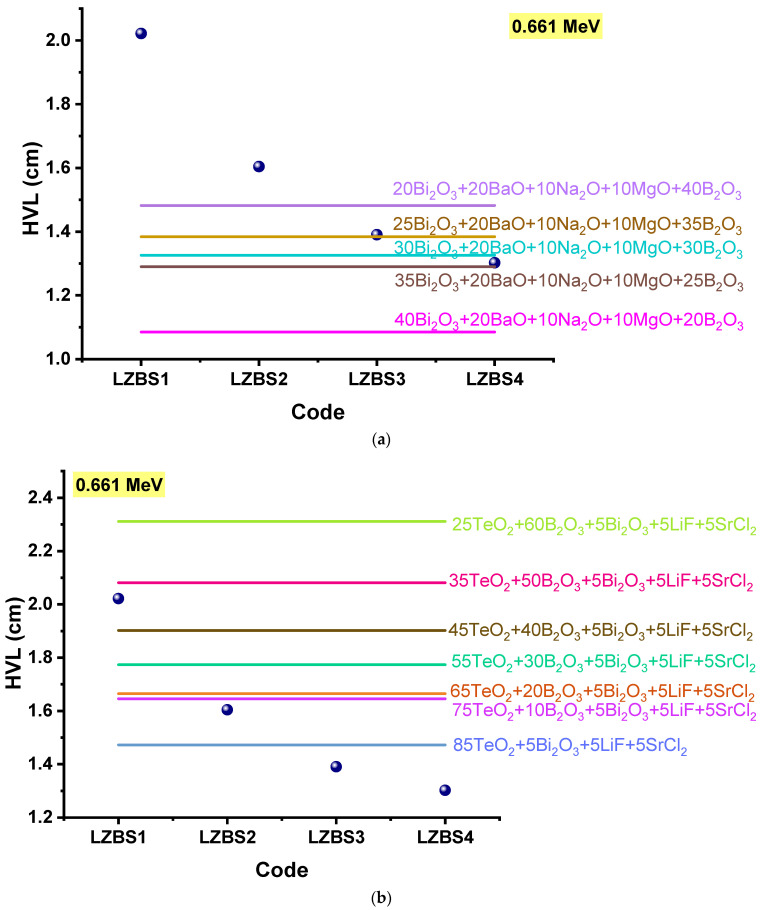

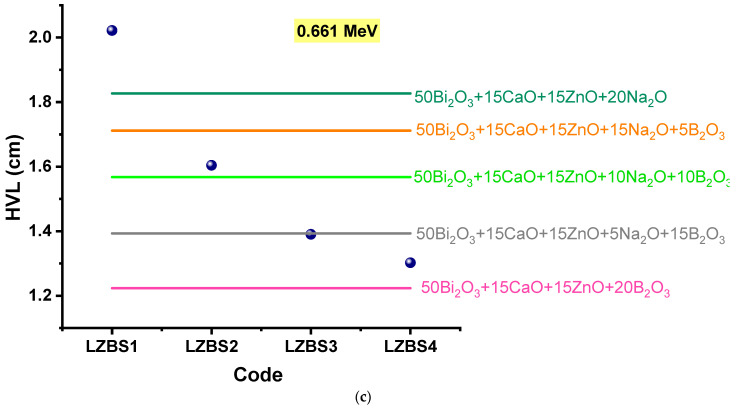
(**a**) Comparison of the HVL of the LZBS glasses with that of the Bi_2_O_3_-BaO-Na_2_O-MgO-B_2_O_3_ glass system [35]. (**b**) Comparison of the HVL of the LZBS glass system with that of the TeO2-B_2_O_3_-Bi_2_O_3_-LiF-SrCl2 glass system [36]. (**c**) Comparison of the HVL of the LZBS glass system with that of the Bi_2_O_3_-CaO-ZnO-Na2O-B_2_O_3_ glass system [23].

A comparison of the HVLs of the LZBS glasses with those of other glasses containing Bi_2_O_3_ is shown in Figure 9a–c.

In Figure 9a, the HVLs of the LZBS glasses are compared with those of the Bi_2_O_3_-BaO-Na_2_O-MgO-B_2_O_3_ glass system, whose radiation shielding was investigated by Sayyed et al. [35]. The LZBS3 glass had an HVL of 1.390 cm, which was lower than that of the 20Bi_2_O_3_ + 20BaO + 10Na_2_O + 10MgO + 40B_2_O_3_ glass (1.482 cm) and comparable to that of the 25Bi_2_O_3_ + 20BaO + 10Na_2_O + 10MgO + 35B_2_O_3_ glass (1.384 cm). The LZBS4 glass, having the lowest HVL in this study, had a value comparable to that of the 35Bi_2_O_3_ + 20BaO + 10Na_2_O + 10MgO + 25B_2_O_3_ glass (1.290 cm).

In Figure 9b, the HVLs of the LZBS glasses are compared with those of the tellurite radiation-shielding glasses studied by Al-Hadeethi et al. [36]. The HVL values obtained at 0.661 MeV of this tellurite glass system were as follows: 2.311, 2.081, 1.902, 1.773, 1.664, 1.645, and 1.472 cm for (25 + x)TeO_2_-(60-x)B_2_O_3_-5Bi_2_O_3_-5LiF-5SrCl_2_, where x = 0–60 mol%. It can be seen that the LZBS glass system fared well in terms of the HVL parameter, as the LZBS4 glass had the lowest HVL (1.302 cm) among all of the glasses in the compared tellurite glass system.

Figure 9c compares the HVLs of the LZBS glasses with those of the Bi_2_O_3_-Na_2_O-ZnO-CaO-B_2_O_3_ radiation-shielding glasses studied by Abouhaswa and Kavaz [23]. The LZBS4 glass with the 30Li_2_O-20ZnO-40Bi_2_O_3_-10SiO_2_ composition had an HVL value (1.302 cm) lower than that of the 50Bi_2_O_3_ + 15CaO + 15ZnO + 20Na_2_O (1.826 cm), 50Bi_2_O_3_ + 15CaO + 15ZnO + 15Na_2_O + 5B_2_O_3_ (1.711 cm), 50Bi_2_O_3_ + 15CaO + 15ZnO + 10Na_2_O + 10B_2_O_3_ (1.567 cm), and 50Bi_2_O_3_ + 15CaO + 15ZnO + 5Na_2_O + 15B_2_O_3_ (1.39 cm) glasses, reflecting its potential for radiation shielding.

## 4. Conclusions

We aimed to study the mechanical and radiation-shielding features of the lithium zinc bismuth silicate glass system using the Makishima–Mackenzie model and Phys-X software. We investigated the influence of Bi_2_O_3_ on the elastic moduli and found that *E* decreased from 46 to 31 GPa, *K* decreased from 27 to 14 GPa, *S* decreased from 19 to 14 GPa, and *L* decreased from 52 to 32 GPa as Bi_2_O_3_ was substituted for SiO_2_. Bi_2_O_3_ not only affected the elastic moduli but also affected the radiation-shielding parameters. The MAC and LAC increased when Bi_2_O_3_ was replaced by SiO_2_. The LZBS1 sample with the lowest amount of Bi_2_O_3_ had the lowest MAC. The ratio between the TVLs of LZBS1 and LZBS4 samples was found to be higher than 1, which suggests that the TVL value of the LZBS1 sample was higher than that of the LZBS4 sample. This ratio was 1.97 at 0.284 MeV and 1.86 at an energy of 0.347 MeV, and it decreased to 1.55 at an energy of 0.662 MeV. Z_eff_ gradually increased as Bi_2_O_3_ content increased from 10 to 40 mol% for a given energy, which indicates a higher photon-interaction probability at higher concentrations of Bi_2_O_3_. The results from this study show that high-density glass samples have a positive effect on the shielding of materials, and the higher the density of the glass, the better the radiation-shielding effect. We compared the HVLs for the selected glasses with those of other glass systems and found that the LZBS3 glass had an HVL of 1.390 cm, which was lower than that of the 20Bi_2_O_3_ + 20BaO + 10Na_2_O + 10MgO + 40B_2_O_3_ glass (1.482 cm) and comparable to that of the 25Bi_2_O_3_ + 20BaO + 10Na_2_O + 10MgO + 35B_2_O_3_ glass (1.384 cm).

## Figures and Tables

**Table 1:** Density (ρ), molar volume (V_m_), packing density (V_t_), and mechanical properties of the selected glasses.

Glass Composition [30]	Glass Code	ρ (g/cm^3^) [30]	V_m_ (cm^3^/mol) [30]	V_t_	E	K	S	*L* (GPa)	H (GPa)
30Li_2_O-20ZnO-10Bi_2_O_3_-40SiO_2_	LZBS1	3.74	25.65	0.49	46	27	19	52	3.55
30Li_2_O-20ZnO-20Bi_2_O_3_-30SiO_2_	LZBS2	4.40	30.99	0.44	40	21	17	43	3.48
30Li_2_O-20ZnO-30Bi_2_O_3_-20SiO_2_	LZBS3	4.90	36.15	0.41	35	18	15	38	3.39
30Li_2_O-20ZnO-40Bi_2_O_3_-10SiO_2_	LZBS4	5.12	42.55	0.38	31	14	14	32	3.32

## Data Availability

Not applicable.
